# Spatial and Temporal Variability in Trihalomethane Concentrations in the Bromine-Rich Public Waters of Perth, Australia

**DOI:** 10.3390/ijerph17197280

**Published:** 2020-10-05

**Authors:** Jessica Stanhope, Gael Davidson, Kimberley McAuley, Angus Cook, Philip Weinstein

**Affiliations:** 1 School of Biological Sciences, The University of Adelaide, Adelaide, SA 5005, Australia; philip.weinstein@adelaide.edu.au; 2 School of Allied Health Science and Practice, The University of Adelaide, Adelaide, SA 5005, Australia; 3 School of Population and Global Health, The University of Western Australia, Perth, WA 6009, Australia; gael.davidson@research.uwa.edu.au (G.D.); kimberley.mcauley@uwa.edu.au (K.M.); angus.cook@uwa.edu.au (A.C.); 4 Discipline of Paediatrics, The University of Western Australia, Perth, WA 6009, Australia; 5 School of Public Health, The University of Adelaide, Adelaide, SA 5005, Australia

**Keywords:** trihalomethane, temporal variation, spatial variation, public health, water quality, water supply

## Abstract

High concentrations of trihalomethanes (THMs) in public water supplies potentially pose a health hazard, but exposure assessment remains a complex task. To interpret research findings and monitoring data for THMs, it is important to evaluate spatial and temporal variations in both total THM and the individual constituent compounds (including brominated species). We therefore aimed to determine the concentrations, and spatial and temporal variability of concentrations, of THMs public water supplies in Perth, Western Australia, which is known historically to have high brominated THM concentrations. We analysed water samples from 21 water distribution zones around Perth (including Busselton and Bunbury) across different seasons over a period of two years. A total of 250 samples provided a median total THM of 72 µg/L (range of 0–157 µg/L), which falls well within Australia’s National Health and Medical Research Council guidelines. The concentration of all species, including brominated forms, also fell the World Health Organization’s guidelines. Total THM concentrations were typically higher in spring and summer. A high degree of spatial variability was detected and appears to relate to the source water. Both the temporal and spatial variability in THM concentrations have implications for epidemiological studies, and monitoring.

## 1. Introduction

Trihalomethanes (THMs) are a group of disinfection by-products produced when halogen-based compounds react with anthropogenic contaminants or natural organic matter [1]. Health impacts that have reportedly been associated with exposure to THMs include adverse birth outcomes [2,3,4] and cancer [5,6]. The current evidence is not sufficiently strong to definitively determine the safety of THMs, with inconsistencies in the current evidence base [2,3,4,5,6]. These reported inconsistencies in previous analyses may relate to different cut-points for high versus low THM exposure, different dominant THM species, differences in the exposure assessment (e.g., using THM concentrations for the residential water distribution zones (WDZs) or individual exposure estimates), and differences in the confounders accounted for in the risk estimates. Further research is required to better understand the potential role of THM exposure in adverse health outcomes, and to set appropriate safety limits. Any potential risks associated with THM exposure are also a critical issue for the future, given that climate change is predicted to contribute to an average increase in THM concentrations of 39% by 2050 [7]. It is therefore crucial to investigate current THM concentrations and their variability to provide a baseline assessment for future studies, and to guide future data collection methods for ongoing monitoring and research. Such monitoring will be more important in the future with these higher anticipated THM concentrations. To develop a strong evidence base for managing THMs in public water supplies, accurate exposure estimates are required. 

The present study is part of a larger project that builds on a previous registry-based prevalence study conducted in Perth, Australia, where total THM (TTHM) concentration within WDZs was correlated with adverse birth outcomes within these zones [8]. To overcome the limitations in exposure estimation of previous studies, our project utilises individual exposure estimates. This required estimates of THMs within public water supplies, as well as from other water sources (e.g., bottled water [9]). Spatial and temporal variability in THM concentrations may occur both between and within WDZs. Variability may result from differences in the characteristics of the source water (namely soil variability, geology, whether it is surface or groundwater, and concentrations of natural organic matter, dissolved organic matter, and halide ions [10]), concentration of total and residual chlorine [11], season [12,13,14], temperature [11,14], as well as water residence time in WDZ [14], and pipe distance (i.e., from the treatment plant to sampling point) [11,12]. A local appreciation of the temporal and spatial variation in THM concentrations through WDZs also has implications for monitoring THM concentration in public water supplies. Furthermore, these exposure estimates should investigate both TTHMs and distinct THM species, as they each have different ‘safe’ limits [15,16]. The differences in the safe limits relate to the different effects that individual THM species may have on biological tissues, with, for example, chloroform being cytotoxic, and brominated THMs identified as mutagenic, cytotoxic, and genotoxic [17]. To optimise both THM monitoring and studies on health effects, the spatial and temporal variability of THM concentrations should first be established. 

Although the spatial and temporal variability of THM concentrations in public water supplies has been investigated previously [10,11,12,13,14], these studies have not been done in locations where brominated THMs (BrTHMs) account for >90% of TTHMs, such as in Perth, Australia [18]. The Swan Coastal Plain of Western Australia, including the metropolitan area of Perth, provides an ideal site for such an investigation due to high concentrations of BrTHMs in treated surface water, groundwater, and mixed water [19]. Bromide, a precursor to BrTHMs, may occur naturally in groundwater from seawater intrusion, or due to run-off with soil which has been treated with brominated pesticides [20,21]. Since 2006, Perth has additionally been supplied with desalinated seawater, which can also contain high concentrations of BrTHMs [22]. 

The purpose of this study was therefore to determine the concentrations, and temporal and spatial variability in concentrations of THMs (TTHMs, BrTHMs, chloroform, bromoform, bromodichloromethane (BDCM), and dibromochloromethane (DBCM)), across metropolitan Perth, Bunbury, and Busselton (Western Australia), to provide the necessary background for accurately assessing exposure to THM concentrations in bromine-rich public water supplies, both for monitoring purposes and epidemiological studies. 

## 2. Materials and Methods 

### 2.1. Water Sampling and Analysis

**Figure 1:**
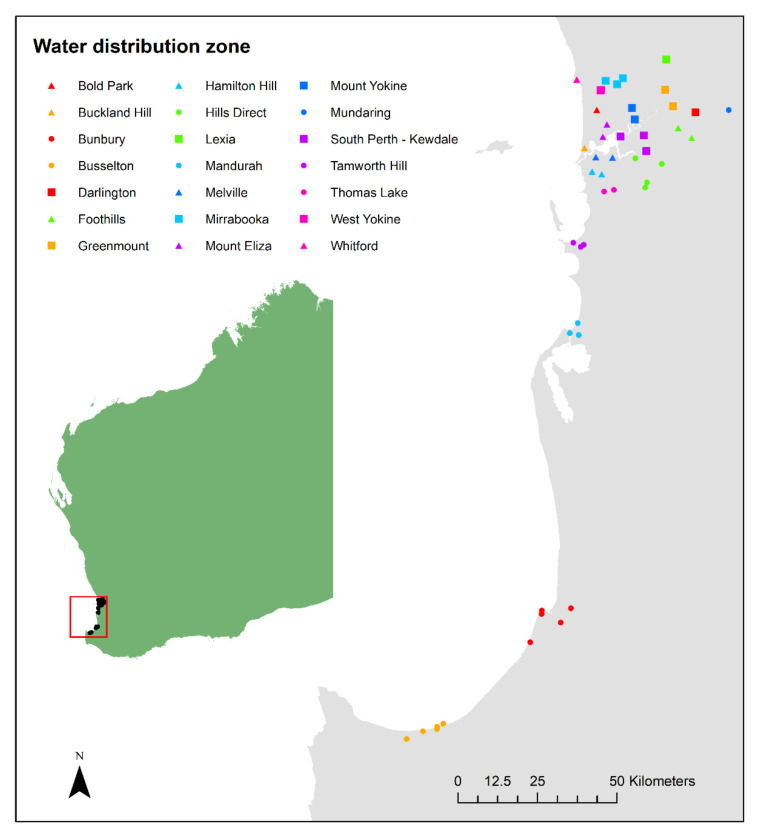
Map of the water sampling sites. Circles indicate sampling sites within water distribution zones (WDZs) with total trihalomethane (TTHM) concentrations in the lowest tertile, triangles indicate sampling sites within WDZs with TTHM concentrations in the middle tertile, and squares indicate WDZs with TTHM concentrations in the highest tertile. (Source: Jessica Stanhope, developed using ArcGIS 10 6 Arc Map).

Water sampling was undertaken across three time points in 2012 and 2013: summer (February), winter (June and August in 2012, and July in 2013), and spring (November). The greater Perth metropolitan region was separated into WDZs based on the historical locations of the supply network to each group of suburbs. Sampling occurred at 1–5 sites within each of the 21 WDZs studied across Perth and in the regional centres of Bunbury and Busselton, resulting in a total of 250 samples (between 6 and 22 for each WDZ; Figure 1). 

Samples were collected from publicly accessible taps in highly populated areas, including drinking fountains, outdoor taps close to picnic areas in public parks, and cold water taps from public toilets, public parks, popular restaurants, and private homes. Sampling sites were chosen where the taps were receiving frequent use, thereby limiting the storage time of water within the pipe network system.

Each water sample (in duplicate) was collected in a 50mL glass vial, which contained the quenching agent, sodium thiosulphate, to prevent further THM formation in the vial. Prior to sampling, each tap was flushed for a timed period of two minutes before collection of the water sample. Each vial was filled to maximum capacity to prevent formation of air bubbles and subsequent loss of THMs to volatilisation. Samples were stored on ice in an insulated container and delivered to MPL Laboratories in Perth. All samples were collected within an 8 h period, kept at 4°C, and analysed within 24–36 h of sampling.

Each sample was analysed for THM concentration (in micrograms per litre) and for the four individual THM species: chloroform, bromoform, BDCM, and DBCM at a National Association of Testing Authorities, Australia (NATA) accredited laboratory [23]. The THM analysis used the “purge and trap” technique for sample concentration and delivery, followed by gas chromatography with mass spectrometric detection (GC/MS) for the separation and quantification of each of the four analyses, as per the United States Environmental Protection Agency (U.S. EPA) 5030C method [24].

### 2.2. Data Analysis

Descriptive statistics were used to summarise the variability of TTHMs, BrTHMs, and their distinct species (chloroform, bromoform, BDCM, and DBCM) over the sampling period. Temporal variability (across years and seasons) was analysed using the Kruskal–Wallis test, as the assumptions of analysis of variance were not met and could not be rectified with data transformation. A 5% level of significance was used. All analyses were conducted in Stata 15 [25]. The raw data are available (doi:10.25909/5d9c14798186b).

## 3. Results

The median TTHM concentration across all time points and WDZs was 72 µg/L, with the highest recorded concentration being 157 µg/L. Brominated THMs predominated, accounting for 97.59% of THMs overall. The highest concentrations of the individual THM species were 62 µg/L for chloroform, 81 µg/L for bromoform, 51 µg/L for BDCM, and 69 µg/L for DBCM (Table 1; Supplementary Materials). 

### 3.1. Temporal Variability

The temporal variability was first examined between the two years, with no significant differences detected in the concentrations of TTHMs, BrTHMs, or the four individual THM species. The second temporal element examined was the seasonal pattern for winter, spring, and summer. The concentrations of TTHMs, BrTHMs, bromoform, and DBCM appear highest in summer, in terms of the mean, median, and maximum concentrations. Chloroform and BDCM concentrations appear highest in spring (Table 2). However, comparisons of concentrations across the three time periods using the Kruskal–Wallis test only achieved statistical significance (*p* < 0.05) for chloroform and bromoform. Statistical analyses were not conducted to compare seasons within WDZs, owing to the small number of samples following stratification, although for most sites, THM concentrations peaked in summer and were lowest in winter (Supplementary Materials).

### 3.2. Spatial Variability 

Spatial variability (between WDZs) was detected for all estimated THM concentrations. Median concentrations within WDZs ranged from 4 µg/L (Busselton) to 112 µg/L (South Perth—Kewdale) for TTHMs; 4 µg/L (Busselton) to 110 µg/L (South Perth—Kewdale) for BrTHMs; 2.5 µg/L (Busselton) to 55 µg/L (Mount Eliza) for bromoform; 0 µg/L (Bunbury and Busselton) to 28 µg/L (Lexia) for BDCM; 1 µg/L (Busselton) to 46 µg/L (South Perth—Kewdale) for DBCM; and from 0 µg/L (Mundaring, Busselton, and Bunbury) to 11 µg/L (Lexia) for chloroform. The concentrations of THMs were typically higher in the northern regions of Perth, and lower in the southern regions of Perth and regional centres (Busselton and Bunbury; both of which are located south of Perth; Table 2 and Figure 1). This pattern was however less consistent when considering the percentage of TTHMs comprised of BrTHMs (Supplementary Materials). There was also some degree of spatial variability within selected WDZs, where multiple sites were sampled, although the ranges detected were typically less than 30 µg/L (Supplementary Materials).

## 4. Discussion

We explored the overall concentrations, as well as the spatial and temporal variability of THM concentrations, in public water supplies, where a predominance of BrTHMs has previously been reported [19]. A high degree of variability of THM concentrations across metropolitan Perth and the two regional centres of Bunbury and Busselton were detected, with implications for both interpreting and conducting exposure assessments in public health research, as well as safety monitoring. 

### 4.1. Temporal Variability

Temporal variability was considered between the two sampling years (2012 and 2013) and three seasons (summer, winter, and spring). There were no significant differences in THM concentrations between the two sampling years, but seasonal differences were detected for chloroform and bromoform. Chloroform typically peaked in spring and was lowest in winter, whereas bromoform typically peaked in summer and was lowest in spring. However, these findings were not necessarily generalisable across WDZs. Concentrations of THMs are known to vary with season [12,13,14], consistent with our study findings. Reasons for seasonal variation in THM concentrations may include temperature differences [11,14], which are known to influence THM concentrations, as well as seasonal changes in water usage which may impact upon water residence times that also affect THM concentrations [14]. Because of issues with access to water sources, not all of the same sites in the same WDZs were available at all sampling events. This sampling variability was unavoidable and may have resulted in a degree of variability in THM concentrations within the WDZs, which could in turn have influenced our findings. Nonetheless, our results indicate that when estimating THM exposures, the temporal variability of THM concentrations in public water supplies should be considered. 

### 4.2. Spatial Variability

Our findings suggest a relationship between latitude and THM concentrations that was largely evident across the species of THMs, as well as total THMs and BrTHMs. Supplies in northern regions had higher THM concentrations compared with southern parts of Perth, and the two southern regional centres (Figure 1). This general pattern was also evident in a report on the 2002/2003 financial year for TTHMs (although the species of THMs were not reported) [19], and appears to have persisted despite changes to Perth’s Integrated Water Supply over the last 10 years, with greater mixing of water sources and the introduction of seawater desalination in 2006 [26]. As of 2017–2018, 48% of Perth’s Integrated Water Supply was from desalinated seawater, 40% from groundwater, 10% from surface water, and 2% groundwater replenishment [27]. By comparison, in 2005, 55% of Perth’s water was sourced from groundwater [19,28]. Most of Perth’s groundwater supplies are situated in the northern areas, whereas southern areas tend to be surface water (and more recently, desalinated). An exception to this pattern is Mundaring, which is supplied by surface water, accounting for the lower THM concentrations in this WDZ compared with those at a similar latitude. The water source can influence the formation of THMs as a result of different concentrations of natural organic matter [10]. Typically, treated surface water has higher concentrations of THMs than groundwater because it contains higher concentrations of precursors for disinfection by-products, including natural organic matter [29], although this comparison depends on the type of aquifer. Perth’s groundwater has historically been extracted from a large, shallow, unconfined sandy aquifer system located approximately 25 kilometres north of the City of Perth [30]. These characteristics may explain the relatively high THM concentrations in the northern areas in our study, as well as previous studies into THM concentrations in Perth’s water supplies [19]. The sandy nature of the system, where soils have low water and nutrient retention, also increases the contamination risk [30]. Given the aquifer system is also shallow and unconfined, it may potentially be contaminated by run-off [31]. Our findings indicate that common assumptions regarding the differences in THM concentrations between WDZs supplied by surface and groundwater must consider the type of aquifer from which the water is sourced. Monitoring for water quality should focus particularly on the WDZs where THM concentrations are typically higher, and therefore, more likely to exceed safe limits—in this case, in the northern WDZs of Perth.

While within-WDZ variability of THM concentrations was not the focus of the present study, data from multiple sites within particular WDZs suggest that a relatively small degree of variability of THM concentrations may occur. The variability detected may relate to water residence time in WDZ [14], and pipe distance (i.e., from treatment plant to sampling point) [11,12]. For the most part, the range of values (within-season) did not exceed 30 µg/L. This is a difference that is unlikely to have major public health implications, particularly given that maximum values were still well within safe limits. 

### 4.3. Water Safety

All water samples taken had concentrations of TTHMs within the limits (250 µg/L) suggested by the National Health and Medical Research Council (NHMRC) [32] in Australia, and chloroform (300 µg/L), bromoform (100 µg/L), BDCM (60 µg/L), and DBCM (100 µg/L) concentrations within the limits suggested by the World Health Organization (WHO) [16]. The WHO and NHMRC guidelines regarding THM concentrations are typically more relaxed than those in some countries [15]. There is wide variation internationally in the ‘safe’ limits for THM concentrations in drinking water, which suggests that evidence-based approaches to setting such limits have not necessarily been adopted. For TTHMs, the limits range from 25 to 250 µg/L, while the ranges of limits are 16–80 µg/L for BDCM, 60–150 µg/L for DBCM, 80–100 µg/L for bromoform, and 60–300 µg/L for chloroform [15]. Using the most conservative (i.e., lowest) THM concentration limits, the median concentrations exceed the safe limits for TTHMs in 19/21 WDZs, 2/21 for BDCM, and no WDZs for DBCM, chloroform, or bromoform. Although the current concentrations for TTHMs are within the current limits in Australia, almost all WDZs could be interpreted as unsafe by international standards, indicating a potential human health risk. As the evidence base is strengthened regarding THM exposure and human health outcomes, these limits will have to be revised internationally, such that an evidence-based approach is adopted. With a 39% increase in THM concentrations predicted by 2050 [7] due to climate change, ongoing monitoring and strategies to further reduce THM concentrations will be necessary.

**Figure 2:**
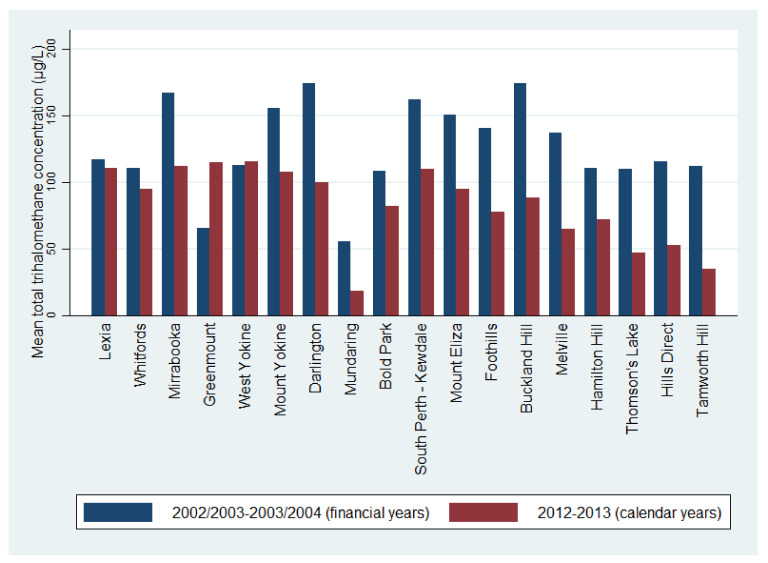
Mean total trihalomethane concentrations (µg/L) from 2002/2003 to 2003/2004 financial years (July–June) [19] and 2012–2013 calendar years (January–December) by water distribution zone. Only water distribution zones with data available at both time points are reported.

Interestingly, the mean concentrations of TTHMs by WDZ detected in our study are lower than those previously reported for the 2002/2003–2003/2004 financial years [19] (Figure 2). A reduction in maximum TTHM concentrations was also observed, with the maximum TTHM concentrations ranging from 68 to 227 µg/L in 2002/2003 and 31 to 157 µg/L in 2012–2013 (for comparable WDZs). These findings reflect mean reductions of between 17 and 82 µ/L, representing decreases of 13 to 65%. These changes in TTHM concentrations may relate to the changes to Perth’s Integrated Water Supply Scheme which shifted to the introduction of seawater desalination [26], accounting for 48% of the water supply in 2017–2018 [27]. In 2017–2018, 40% of Perth’s Integrated Water Supply Scheme was sourced from groundwater, 10% from surface water and 2% groundwater replenishment [27]. Our findings suggest that these changes in water supply have been effective in reducing TTHM concentrations in Perth, despite a higher water demand because of an increasing population [33] and a reduction in rainfall [34]. Even with the less stringent NHMRC guidelines in Australia compared to other countries with lower TTHM safety limits, these supply changes and TTHM reductions are consistent with protecting the public from any potentially adverse effects of exposure to high THMs in public water supplies. 

## 5. Conclusions

There is a degree of spatial and temporal variation in THM concentrations in Perth and the surrounding regional centres of Busselton and Bunbury. The magnitude of temporal variation, while statistically significant, was relatively small. Nevertheless, it is recommended that water quality monitoring should focus on those seasons (notably spring and summer, based on our results) when THM concentrations are most likely to exceed safe limits. A high degree of spatial variability was detected and appears to relate to the source water. Importantly, there have been substantial decreases in the TTHM concentrations in Perth in the last 10 years, which may relate to the introduction of seawater desalination and greater mixing of water sources. Overall, our findings contribute to the necessary background understanding required to accurately assess exposure. When considering the most conservative TTHM concentration safe limits for drinking water internationally, 19 of the 21 WDZs in Perth exceed these limits. There is an urgent need for future research into the human health effects of exposure to THMs in drinking water to inform evidence-based guidelines regarding safe limits. Ongoing vigilance and targeted testing are recommended in the context of predicted future increases in THM concentrations with climate change. Water departments thus have an ongoing responsibility to work towards further reducing THM concentrations in public water supplies. 

## Figures and Tables

**Table 1:** Summary of the concentrations (µg/L) of total trihalomethanes, total brominated trihalomethanes, and the four species of trihalomethanes across the 21 water distribution zones (ordered from north to south).

**Water Distribution Zone** **(Number of Samples)**	**Total Trihalomethanes** **(µg/L)**		**Chloroform** **(µg/L)**		**Total Brominated Trihalomethanes (µg/L)**		**Bromoform** **(µg/L)**		**Bromodichloromethane (µg/L)**		**Dibromochloromethane** **(µg/L)**	
	**Median (IQR)**	**Maximum**	**Median (IQR)**	**Maximum**	**Median (IQR)**	**Maximum**	**Median (IQR)**	**Maximum**	**Median (IQR)**	**Maximum**	**Median (IQR)**	**Maximum**
Lexia (*n* = 6)	111.0 (97.0–116.0)	142.0	11.0 (8.0–51.0)	62.0	84.0 (64.0–103.0)	130.0	24.0 (2.0–38.0)	2.0–38.0	28.0 (20.0–37.0)	41.0	38.5 (21.0–44.0)	59.0
Whitfords (*n* = 6)	94.5 (88.0–111.0)	112.0	6.5 (3.0–7.0)	8.0	92.0 (81.0–103.0	105.0	37.5 (24.0–44.0)	24.0–44.0	14.0 (11.0–20.0)	23.0	38.0 (37.0–48.0)	53.0
Mirrabooka (*n* = 13)	106.0 (83.0–133.0)	157.0	7.0 (5.0–9.0)	27.0	97.0 (81.0–124.0)	153.0	31.0 (22.0–38.0)	22.0–38.0	24.0 (17.0–26.0)	51.0	45.0 (33.0–58.0)	69.0
Greenmount (*n* = 12)	112.0 (102.5–124.5)	157.0	3.0 (3.0–4.5)	10.0	109.0 (98.5–120.0)	147.0	54.0 (50.0–57.0)	50.0–57.0	14.5 (13.0–17.0)	24.0	43.5 (40.5–49.0)	58.0
West Yokine (*n* = 11)	108.0 (102.0–131.0)	152.0	4.0 (3.0–8.0)	9.0	105.0 (99.0–127.0)	143.0	52.0 (44.0–55.0)	44.0–55.0	14.0 (12.0–20.0)	24.0	43.0 (39.0–51.0)	55.0
Mount Yokine (*n* = 12)	105.0 (99.0–113.0)	137.0	3.0 (3.0–4.5)	9.0	102.0 (95.5–110.0)	128.0	49.5 (45.0–52.5)	45.0–52.5	13.5 (12.5–17.0)	22.0	41.5 (39.5–45.0)	50.0
Darlington (*n* = 6)	100.0 (87.0–115.0)	122.0	2.0 (2.2–2.0)	8.0	95.0 (85.0–115.0)	120.0	52.0 (36.0–66.0)	36.0–66.0	9.0 (8.0–10.0)	23.0	33.5 (30.0–40.0)	43.0
Mundaring (*n* = 6)	15.5 (15.0–21.0)	31.0	0.0 (0.0–1.0)	1.0	15.5 (14.0–21.0)	30.0	8.5 (7.0–11.0)	7.0–11.0	2.0 (2.0–2.0)	4.0	5.5 (5.0–8.0)	11.0
Bold Park (*n* = 6)	79.0 (68.0–102.0)	119.0	1.5 (1.0–3.0)	9.0	78.0 (67.0–99.0)	110.0	38.0 (30.0–45.0)	30.0–45.0	8.0 (5.0–11.0)	27.0	30.5 (20.0–38.0)	53.0 *(continued)*
South Perth— Kewdale (*n* = 19)	112.0 (105.0–131.0)	155.0	5.0 (3.0–8.0)	10.0	110.0 (96.0–122.0)	149.0	50.0 (36.0–59.0)	36.0-59.0	16.0 (13.0–20.0)	25.0	46.0 (36.0–52.0)	57.0
Mount Eliza (*n* = 14)	94.5 (74.0–108.0)	151.0	2.0 (1.0–3.0)	6.0	93.5 (72.0–105.0)	145.0	55.0 (45.0–67.0)	45.0–67.0	7.0 (4.0–12.0)	21.0	29.0 (16.0–32.0)	53.0
Foothills (*n* = 12)	85.0 (58.5–96.5)	140.0	2.0 (1.0–3.5)	13.0	84.0 (57.0–90.5)	127.0	36.0 (25.5–48.5)	25.5–48.5	7.0 (5.5–12.0)	34.0	27.5 (20.0–38.0)	58.0
Buckland Hill (*n* = 6)	91.5 (58.0–111.0)	125.0	1.0 (1.0–2.0)	6.0	91.0 (57.0–109.0)	119.0	49.5 (36.0–60.0)	36.0–60.0	6.5 (4.0–8.0)	20.0	28.5 (17.0–36.0)	49.0
Melville (*n* = 13)	59.0 (43.0–87.0)	114.0	2.0 (1.0–3.0)	7.0	57.0 (43.0–87.0)	109.0	34.0 (16.0–47.0)	16.0–47.0	6.0 (5.0–10.0)	17.0	22.0 (18.0–26.0)	45.0
Hamilton Hill (*n* = 12)	69.5 (43.0–87.0)	110.0	2.0 (1.0–2.0)	7.0	67.5 (51.5–79.5)	108.0	40.5 (28.0–48.0)	28.0–48.0	6.0 (5.0–9.0)	16.0	20.5 (18.0–27.0)	39.0
Thomson’s Lake (*n* = 12)	48.0 (42.0–56.0)	83.0	1.0 (0.0–2.0)	4.0	46.0 (41.5–55.5)	81.0	27.0 (24.5–34.5)	24.5–34.5	4.0 (3.0–5.0)	8.0	13.5 (11.5–18.0)	27.0
Hills Direct (*n* = 18)	49.5 (34.0–69.0)	112.0	2.0 (1.0–6.0)	12.0	45.5 (32.0–59.0)	100.0	19.5 (12.0–23.0)	12.0–23.0	6.5 (4.0–16.0)	28.0	18.0 (14.0–28.0)	50.0
Tamworth Hill (*n* = 15)	34.0 (19.0–46.0)	71.0	1.0 (0.0–2.0)	8.0	34.0 (18.0–44.0)	63.0	11.0 (8.0–13.0)	8.0–13.0	6.0 (3.0–10.0)	19.0	17.0 (8.0–20.0)	31.0
Mandurah (*n* = 15)	31.0 (17.0–56.0)	77.0	1.0 (0.0–4.0)	9.0	31.0 (16.0–52.0)	68.0	10.0 (7.0–14.0)	7.0–14.0	5.0 (3.0–11.0)	21.0	17.0 (6.0–23.0)	34.0 *(continued)*
Bunbury (*n* = 22)	7.0 (6.0–27.0)	55.0	0.0 (0.0–0.0)	2.0	7.0 (6.0–26.0)	55.0	5.0 (3.0–12.0)	3.0-12.0	0.0 (0.0–2.0)	7.0	2.0 (2.0–10.0)	18.0
Busselton (*n* = 14)	4.0 (1.0–6.0)	16.0	0.0 (0.0–0.0)	0.0	4.0 (1.0–6.0)	16.0	2.5 (1.0–3.0)	1.0-3.0	0.0 (0.0–0.0)	2.0	1.0 (0.0–2.0)	4.0

*Notes:* Shading indicates the tertiles based on the median (dark—high; light—medium; no—low). IQR—interquartile range. See Supplementary Materials for further information.

**Table 2:** Summary of the concentrations (µg/L) of total trihalomethanes, total brominated trihalomethanes, and the four species of trihalomethanes across all sampling periods together, and winter, spring, and summer.

		Concentration (µg/L)				Kruskal-Wallis Test
		All Time Periods	Winter	Spring	Summer	
Total trihalomethanes	Mean (SD)	69.32 (43.26)	61.71 (39.43)	69.33 (42.61)	77.67 (46.42)	χ^2^ (2) = 5.28, *p* = 0.071
	Median (IQR)	72.00 (31.00–105.00)	57.00 (21.00–98.00)	76.00 (30.00–104.00)	83.50 (40.50–115.00)	
	Range	0.00–157.00	2.00–155.00	0.00–155.00	0.00–157.00	
Chloroform	Mean (SD)	3.44 (5.89)	2.51 (2.63)	4.01 (4.21)	3.76 (8.86)	χ^2^ (2) = 7.23, *p* = 0.027*
	Median (IQR)	2.00 (0.00–4.00)	2.00 (1.00–3.00)	3.00 (1.00–7.00)	2.00 (0.00–3.00)	
	Range	0.00–62.00	0.00–12.00	0.00–27.00	0.00–62.00	
Total brominated trihalomethanes	Mean (SD)	66.20 (40.94)	59.20 (37.44)	65.32 (40.12)	73.90 (44.06)	χ^2^ (2) = 4.58, *p* = 0.101
	Median (IQR)	67.00 (30.00–102.00)	56.00 (20.00–94.00)	70.50 (30.00–99.00)	81.00 (39.50–109.50)	
	Range	0.00–153.00	2.00–149.00	0.00–145.00	0.00–153.00	
Bromoform	Mean (SD)	30.40 (20.77)	28.70 (18.04)	26.98 (19.55)	35.48 (23.54)	χ^2^ (2) = 6.28, *p* = 0.043*
	Median (IQR)	28.00 (11.00–48.00)	30.00 (10.00–39.00)	22.00 (11.00–47.00)	38.50 (14.00–55.50)	
	Range	0.00–81.00	2.00–78.00	0.00–71.00	0.00–81.00	
Bromodichloromethane	Mean (SD)	10.00 (8.60)	8.44 (7.72)	11.45 (9.41)	10.07 (8.41)	χ^2^ (2) = 5.01, *p* = 0.082
	Median (IQR)	8.00 (3.00–15.00)	5.00 (3.00–12.00)	12.00 (3.00–18.00)	8.00 (5.00–14.00)	
	Range	0.00–51.00	0.00–33.00	0.00–51.00	0.00–41.00	
Dibromochloromethane	Mean (SD)	25.80 (17.19)	22.06 (16.01)	26.89 (17.40)	28.36 (17.65)	χ^2^ (2) = 5.42, *p* = 0.067
	Median (IQR)	24.00 (11.00–40.00)	17.50 (8.00–35.00)	29.50 (10.00–41.00)	27.00 (16.50–40.50)	
	Range	0.00–69.00	0.00–59.00	0.00–61.00	0.00–69.00	

*Notes:* SD—standard deviation; IQR—interquartile range; *p < 0.05, **p < 0.01, ***p < 0.001

## Supplementary Materials

The following are available online at https://www.mdpi.com/1660-4601/17/19/7280/s1, Table S1: Summary of concentrations of trihalomethanes across the 21 water distribution zones (ordered north to south), Figure S1: Seasonal variability in the median total trihalomethane concentrations (µg/L) by water distribution zone, Figure S2: Seasonal variability in the median total brominated trihalomethane concentration (µg/L) by water distribution zone, Figure S3: Seasonal variability in the median chloroform concentration (µg/L) by water distribution zone, Figure S4: Seasonal variability in the median bromoform concentration (µg/L) by water distribution zone, Figure S5: Seasonal variability in the median bromodichloromethane concentrations (µg/L) by water distribution zone, Figure S6: Seasonal variability in the median dibromochloromethane concentrations (µg/L) by water distribution zone, Table S2: Variability of trihalomethane concentrations within water distribution zones. The raw data are available: doi:10.25909/5d9c14798186b
