# Supplementary material for: Spatial and Temporal Variability in Trihalomethane Concentrations in the Bromine-Rich Public Waters of Perth, Australia
Source: Int J Environ Res Public Health. 2020 Oct 5;17(19):7280. doi: 10.3390/ijerph17197280 (PMC7579358; doi:10.3390/ijerph17197280)
Supplement: Supplementary file 1 [file ijerph-17-07280-s001.pdf]

Supplementary Material A: Summary of concentrations of trihalomethanes across the 21 water distribution zones (ordered north to south)

Table S1: Summary of concentrations of trihalomethanes across the 21 water distribution zones (ordered north to south)

Water distribution zone (number of samples)	Total trihalomethanes (µg/L)					Chloroform (µg/L)				
	Mean	Standard deviation	Median	Inter-quartile range	Range	Mean	Standard deviation	Median	Inter-quartile range	Range
Lexia (n=6)	111.0	18.4	111.0	97.0-116.0	89.0-142.0	24.5	25.2	11.0	8.0-51.0	4.0-62.0
Whitfords (n=6)	94.8	16.0	94.5	88.0-111.0	69.0-112.0	5.5	2.4	6.5	3.0-7.0	2.0-8.0
Mirrabooka (n=13)	112.2	30.6	106.0	83.0-133.0	72.0-157.0	8.5	6.2	7.0	5.0-9.0	2.0-27.0
Greenmount (n=12)	115.3	16.9	112.0	102.5-124.5	95.0-157.0	4.1	2.1	3.0	3.0-4.5	3.0-10.0
West Yokine (n=11)	116.4	18.9	108.0	102.0-131.0	97.0-152.0	4.9	2.5	4.0	3.0-8.0	3.0-9.0
Mount Yokine (n=12)	108.3	11.9	105.0	99.0-113.0	98.0-137.0	4.3	2.3	3.0	3.0-4.5	3.0-9.0
Darlington (n=6)	100.2	16.8	100.0	87.0-115.0	77.0-122.0	2.7	2.7	2.0	2.0-2.0	0.0-8.0
Mundaring (n=6)	18.7	6.5	15.5	15.0-21.0	14.0-31.0	0.3	0.5	0.0	0.0-1.0	0.0-1.0
Bold Park (n=6)	82.2	26.2	79.0	68.0-102.0	46.0-119.0	2.7	3.3	1.5	1.0-3.0	0.0-9.0
South Perth – Kewdale (n=19)	110.4	25.9	112.0	105.0-131.0	56.0-155.0	5.0	2.8	5.0	3.0-8.0	0.0-10.0
Mount Eliza (n=14)	94.6	29.6	94.5	74.0-108.0	50.0-151.0	2.1	1.9	2.0	1.0-3.0	0.0-6.0
Foothills (n=12)	78.4	34.3	85.0	58.5-96.5	0.0-140.0	3.4	4.2	2.0	1.0-3.5	0.0-13.0
Buckland Hill (n=6)	88.7	29.1	91.5	58.0-111.0	55.0-125.0	1.8	2.1	1.0	1.0-2.0	0.0-6.0
Melville (n=13)	65.3	30.7	59.0	43.0-87.0	21.0-114.0	2.2	2.1	2.0	1.0-3.0	0.0-7.0
Hamilton Hill (n=12)	71.5	20.3	69.5	53.0-80.5	48.0-110.0	2.3	1.9	2.0	1.0-2.0	1.0-7.0
Thomson's Lake (n=12)	46.6	20.4	48.0	42.0-56.0	8.0-83.0	1.3	1.2	1.0	0.0-2.0	0.0-4.0
Hills Direct (n=18)	52.5	26.7	49.5	34.0-69.0	20.0-112.0	3.4	3.4	2.0	1.0-6.0	0.0-12.0
Tamworth Hill (n=15)	34.8	22.0	34.0	19.0-46.0	3.0-71.0	2.3	3.1	1.0	0.0-2.0	0.0-8.0
Mandurah (n=15)	35.3	26.4	31.0	17.0-56.0	0.0-77.0	2.5	3.3	1.0	0.0-4.0	0.0-9.0
Bunbury (n=22)	14.9	15.5	7.0	6.0-27.0	0.0-55.0	0.1	0.5	0.0	0.0-0.0	0.0-2.0
Busselton (n=14)	4.4	4.2	4.0	1.0-6.0	0.0-16.0	0.0	0.0	0.0	0.0-0.0	0.0-0.0

(continued →)

Note: Colours indicate the tertiles based on the median (red = high, orange = medium, green = low)

Water distribution zone (number of samples)	Total brominated trihalomethanes (µg/L)					Bromoform (µg/L)				
	Mean	Standard deviation	Median	Inter-quartile range	Range	Mean	Standard deviation	Median	Inter-quartile range	Range
Lexia (n=6)	86.5	27.4	84.0	64.0-103.0	54.0-130.0	22.3	18.1	24.0	2.0-38.0	1.0-45.0
Whitfords (n=6)	89.3	15.6	92.0	81.0-103.0	63.0-105.0	34.8	11.8	37.5	24.0-44.0	18.0-48.0
Mirrabooka (n=13)	103.8	28.6	97.0	81.0-124.0	65.0-153.0	33.9	16.0	31.0	22.0-38.0	17.0-76.0
Greenmount (n=12)	111.3	15.5	109.0	98.5-120.0	92.0-147.0	52.1	8.5	54.0	50.0-57.0	36.0-65.0
West Yokine (n=11)	111.5	16.9	105.0	99.0-127.0	94.0-143.0	50.6	9.2	52.0	44.0-55.0	35.0-65.0
Mount Yokine (n=12)	104.1	10.2	102.0	95.5-110.0	94.0-128.0	47.7	6.9	49.5	45.0-52.5	35.0-56.0
Darlington (n=6)	97.5	17.3	95.0	85.0-115.0	75.0-120.0	51.7	18.6	52.0	36.0-66.0	28.0-76.0
Mundaring (n=6)	18.3	6.3	15.5	14.0-21.0	14.0-30.0	9.5	3.1	8.5	7.0-11.0	7.0-15.0
Bold Park (n=6)	79.5	23.9	78.0	67.0-99.0	45.0-110.0	37.5	9.9	38.0	30.0-45.0	24.0-50.0
South Perth – Kewdale (n=19)	105.4	24.8	110.0	96.0-122.0	56.0-149.0	46.5	17.3	50.0	36.0-59.0	14.0-78.0
Mount Eliza (n=14)	92.5	28.2	93.5	72.0-105.0	49.0-145.0	55.6	15.1	55.0	45.0-67.0	29.0-81.0
Foothills (n=12)	75.0	31.5	84.0	57.0-90.5	0.0-127.0	35.8	16.7	36.0	25.5-48.5	0.0-59.0
Buckland Hill (n=6)	86.8	27.8	91.0	57.0-109.0	54.0-119.0	49.5	13.2	49.5	36.0-60.0	34.0-68.0
Melville (n=13)	63.1	29.9	57.0	43.0-87.0	20.0-109.0	32.9	18.5	34.0	16.0-47.0	10.0-58.0
Hamilton Hill (n=12)	69.3	20.8	67.5	51.5-79.5	44.0-108.0	38.8	17.0	40.5	28.0-48.0	11.0-70.0
Thomson's Lake (n=12)	45.3	19.6	46.0	41.5-55.5	8.0-81.0	27.2	11.3	27.0	24.5-34.5	4.0-46.0
Hills Direct (n=18)	49.1	24.0	45.5	32.0-59.0	19.0-100.0	17.9	6.2	19.5	12.0-23.0	10.0-29.0
Tamworth Hill (n=15)	32.5	19.4	34.0	18.0-44.0	3.0-63.0	10.1	4.1	11.0	8.0-13.0	2.0-16.0
Mandurah (n=15)	32.8	23.7	31.0	16.0-52.0	0.0-68.0	10.0	5.9	10.0	7.0-14.0	0.0-18.0
Bunbury (n=22)	14.7	15.2	7.0	6.0-26.0	0.0-55.0	9.1	9.6	5.0	3.0-12.0	0.0-42.0
Busselton (n=14)	4.4	4.2	4.0	1.0-6.0	0.0-16.0	2.9	2.6	2.5	1.0-3.0	0.0-10.0

(continued →)

Note: Colours indicate the tertiles based on the median (red = high, orange = medium, green = low)

Water distribution zone (number of samples)	Bromodichloromethane (µg/L)					Dibromochloromethane (µg/L)				
	Mean	Standard deviation	Median	Inter-quartile range	Range	Mean	Standard deviation	Median	Inter-quartile range	Range
Lexia (n=6)	28.0	10.6	28.0	20.0-37.0	14.0-41.0	36.2	15.8	38.5	21.0-44.0	16.0-59.0
Whitfords (n=6)	14.0	7.5	14.0	11.0-20.0	2.0-23.0	40.5	8.6	38.0	37.0-48.0	29.0-53.0
Mirrabooka (n=13)	23.5	10.8	24.0	17.0-26.0	10.0-51.0	46.4	13.5	45.0	33.0-58.0	30.0-69.0
Greenmount (n=12)	15.2	3.5	14.5	13.0-17.0	11.0-24.0	44.0	7.1	43.5	40.5-49.0	32.0-58.0
West Yokine (n=11)	16.2	4.6	14.0	12.0-20.0	11.0-24.0	44.7	6.4	43.0	39.0-51.0	35.0-55.0
Mount Yokine (n=12)	14.8	3.7	13.5	12.5-17.0	11.0-22.0	41.6	5.2	41.5	39.5-45.0	33.0-50.0
Darlington (n=6)	10.8	6.1	9.0	8.0-10.0	6.0-23.0	35.0	5.6	33.5	30.0-40.0	30.0-43.0
Mundaring (n=6)	2.3	0.8	2.0	2.0-2.0	2.0-4.0	6.5	2.6	5.5	5.0-8.0	4.0-11.0
Bold Park (n=6)	10.7	8.3	8.0	5.0-11.0	5.0-27.0	31.3	13.4	30.5	20.0-38.0	16.0-53.0
South Perth – Kewdale (n=19)	15.8	5.1	16.0	13.0-20.0	7.0-25.0	43.1	10.1	46.0	36.0-52.0	23.0-57.0
Mount Eliza (n=14)	8.9	5.9	7.0	4.0-12.0	2.0-21.0	28.1	12.6	29.0	16.0-32.0	9.0-53.0
Foothills (n=12)	10.8	9.7	7.0	5.5-12.0	0.0-34.0	28.5	14.7	27.5	20.0-38.0	0.0-58.0
Buckland Hill (n=6)	8.2	6.1	6.5	4.0-8.0	4.0-20.0	29.2	12.7	28.5	17.0-36.0	16.0-49.0
Melville (n=13)	7.5	4.5	6.0	5.0-10.0	2.0-17.0	22.7	10.7	22.0	18.0-26.0	7.0-45.0
Hamilton Hill (n=12)	7.4	3.6	6.0	5.0-9.0	4.0-16.0	23.0	7.0	20.5	18.0-27.0	15.0-39.0
Thomson's Lake (n=12)	4.2	2.3	4.0	3.0-5.0	1.0-8.0	14.0	6.7	13.5	11.5-18.0	3.0-27.0
Hills Direct (n=18)	9.5	7.4	6.5	4.0-16.0	2.0-28.0	21.7	13.0	18.0	14.0-28.0	6.0-50.0
Tamworth Hill (n=15)	7.3	6.3	6.0	3.0-10.0	0.0-19.0	15.1	10.0	17.0	8.0-20.0	1.0-31.0
Mandurah (n=15)	7.5	7.1	5.0	3.0-11.0	0.0-21.0	15.3	11.8	17.0	6.0-23.0	0.0-34.0
Bunbury (n=22)	1.1	1.8	0.0	0.0-2.0	0.0-7.0	4.6	4.9	2.0	2.0-10.0	0.0-18.0
Busselton (n=14)	0.3	0.6	0.0	0.0-0.0	0.0-2.0	1.1	1.2	1.0	0.0-2.0	0.0-4.0

(continued →)

Note: Colours indicate the tertiles based on the median (red = high, orange = medium, green = low)

Water distribution zone (number of samples)	Percentage of total trihalomethanes that are brominated				
	Mean	Standard deviation	Median	Inter-quartile range	Range
Lexia (n=6)	78.5	21.5	90.4	55.6-91.6	46.6-96.3
Whitfords (n=6)	94.1	2.7	93.3	92.1-96.9	91.3-97.9
Mirrabooka (n=13)	92.5	4.1	92.2	90.9-96.3	82.6-97.6
Greenmount (n=12)	96.5	1.3	97.2	95.5-97.4	93.6-97.6
West Yokine (n=11)	95.9	1.5	96.4	94.1-97.1	93.6-97.7
Mount Yokine (n=12)	96.2	1.6	97.0	95.4-97.3	92.6-97.4
Darlington (n=6)	97.3	2.7	97.8	97.4-98.4	92.2-100.0
Mundaring (n=6)	98.4	2.8	100.0	96.8-100.0	93.3-100.0
Bold Park (n=6)	97.2	2.6	97.5	97.1-98.5	92.4-100.0
South Perth – Kewdale (n=19)	95.6	2.6	96.1	93.4-97.3	89.5-100.0
Mount Eliza (n=14)	98.1	1.4	98.1	97.3-98.5	95.3-100.0
Foothills (n=12)	96.2	3.4	97.6	95.5-98.0	88.9-100.0
Buckland Hill (n=6)	98.1	1.6	98.2	98.2-98.7	95.2-100.0
Melville (n=13)	96.4	3.1	96.6	95.5-98.2	87.5-100.0
Hamilton Hill (n=12)	96.5	3.3	97.6	96.7-98.5	89.4-99.0
Thomson's Lake (n=12)	97.7	2.1	97.8	95.8-100.0	94.0-100.0
Hills Direct (n=18)	94.4	3.6	95.4	93.1-96.2	85.5-100.0
Tamworth Hill (n=15)	95.4	4.3	95.4	94.1-100.0	88.1-100.0
Mandurah (n=15)	94.6	4.1	94.4	92.9-96.1	88.3-100.0
Bunbury (n=22)	99.6	1.2	100.0	100.0-100.0	95.8-100.0
Busselton (n=14)	100.0	0.0	100.0	100.0-100.0	95.8-100.0

Note: Colours indicate the tertiles based on the median (red = high, orange = medium, green = low)

Supplementary Material B: Seasonal variability in trihalomethane concentrations by water distribution zone

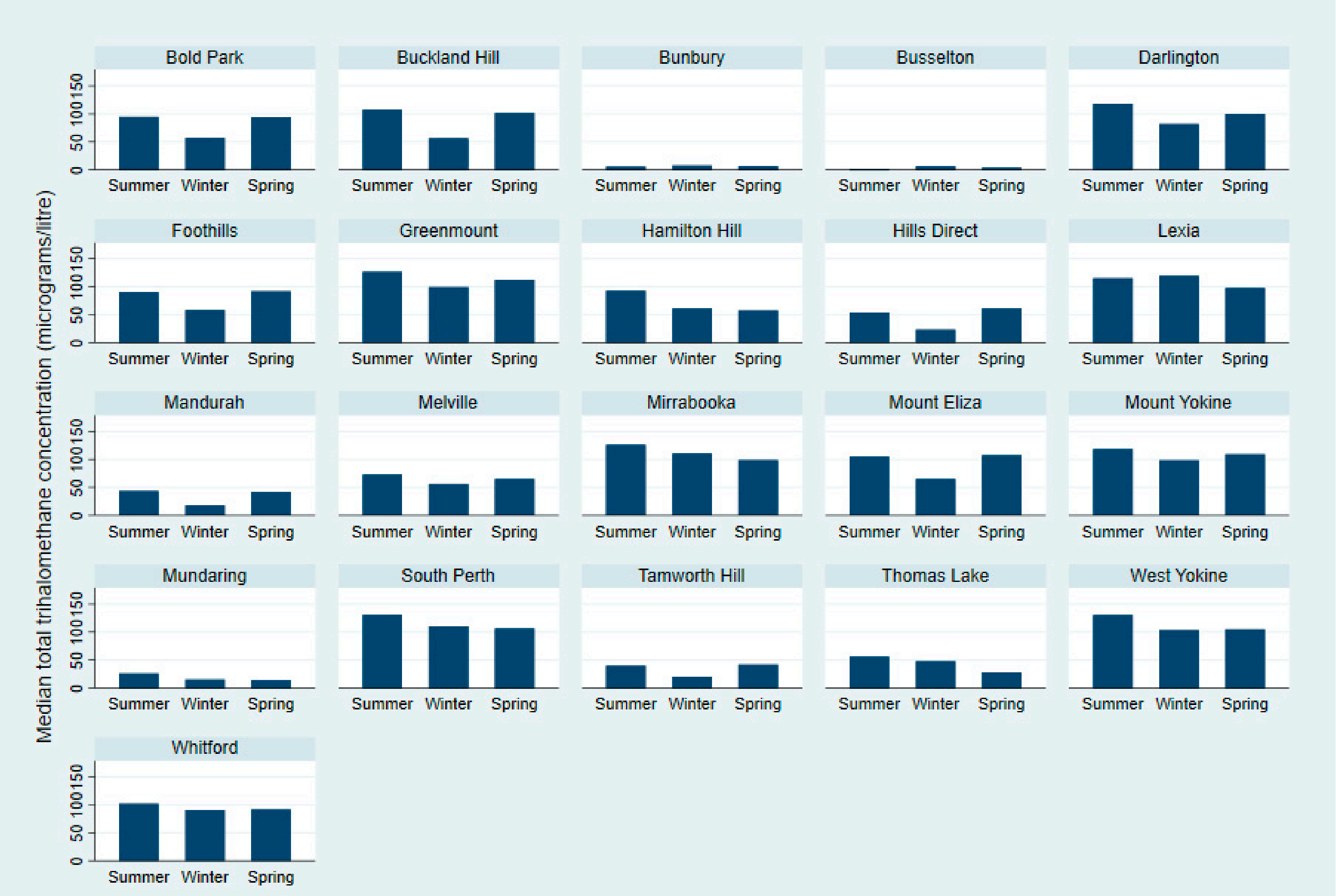

Figure S1: Seasonal variability in the median total trihalomethane concentrations (µg/L) by water distribution zone

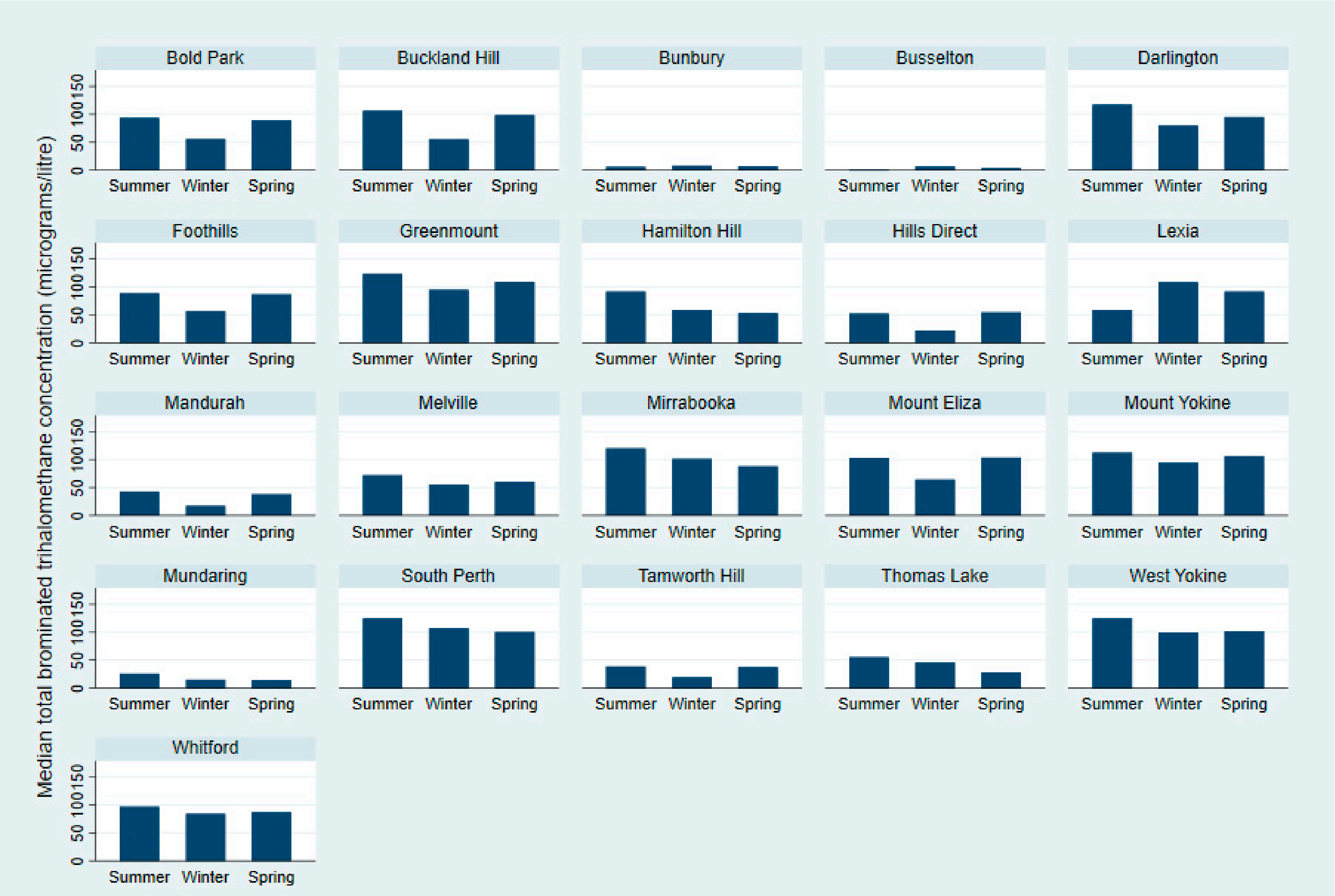

Figure S2: Seasonal variability in the median total brominated trihalomethane concentrations (μg/L) by water distribution zone

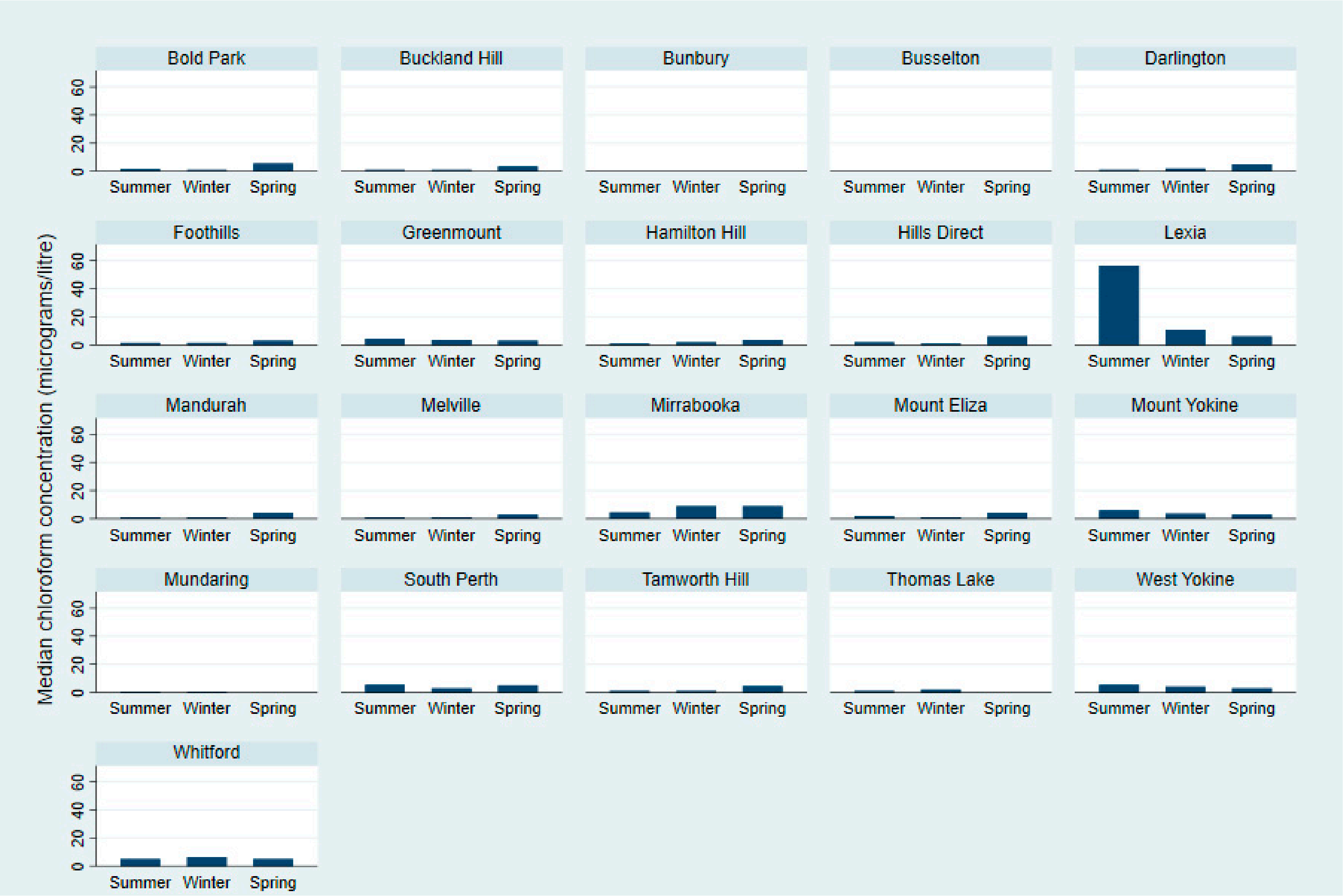

Figure S3: Seasonal variability in the median chloroform concentrations (µg/L) by water distribution zone

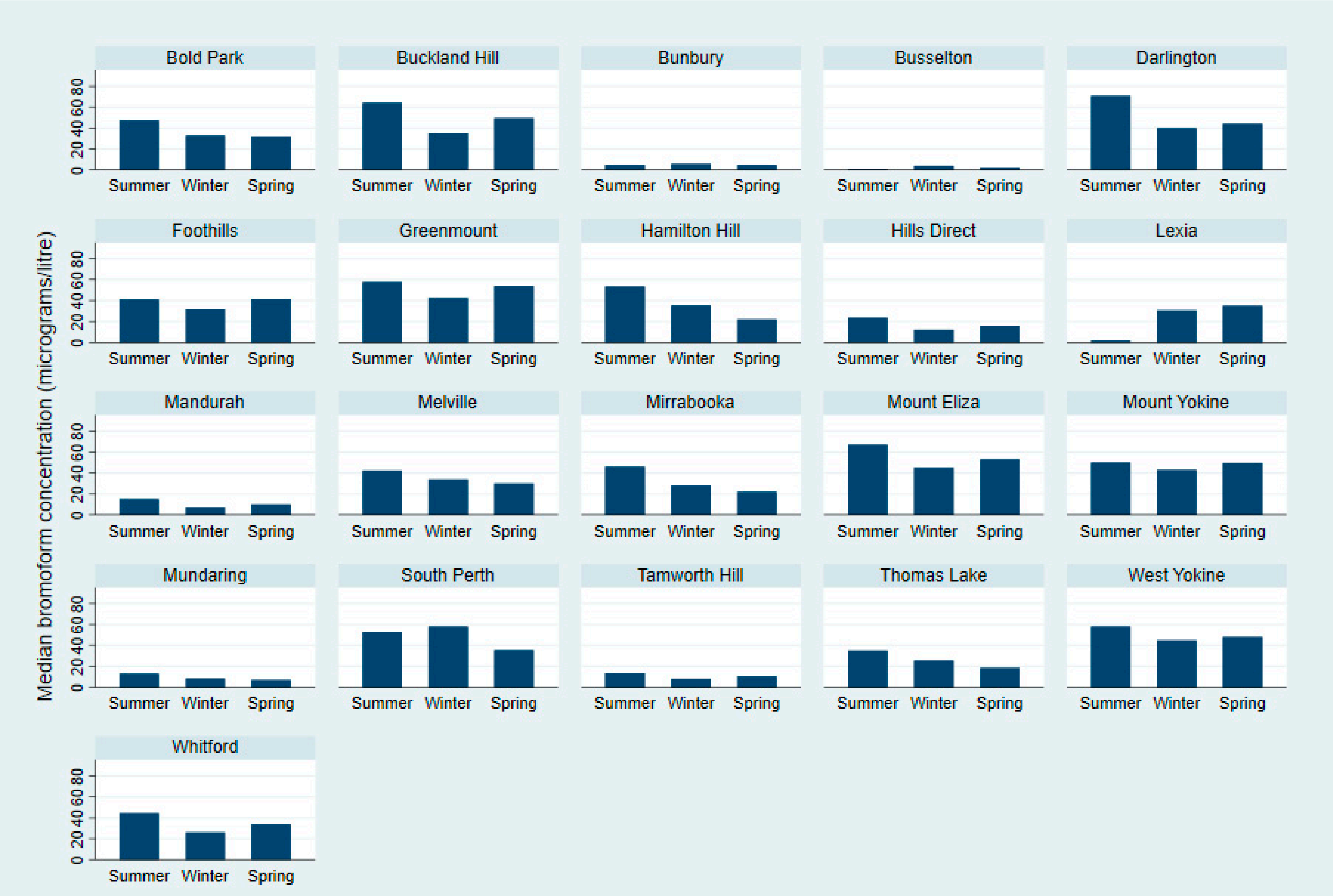

Figure S4: Seasonal variability in the median bromoform concentrations (μg/L) by water distribution zone

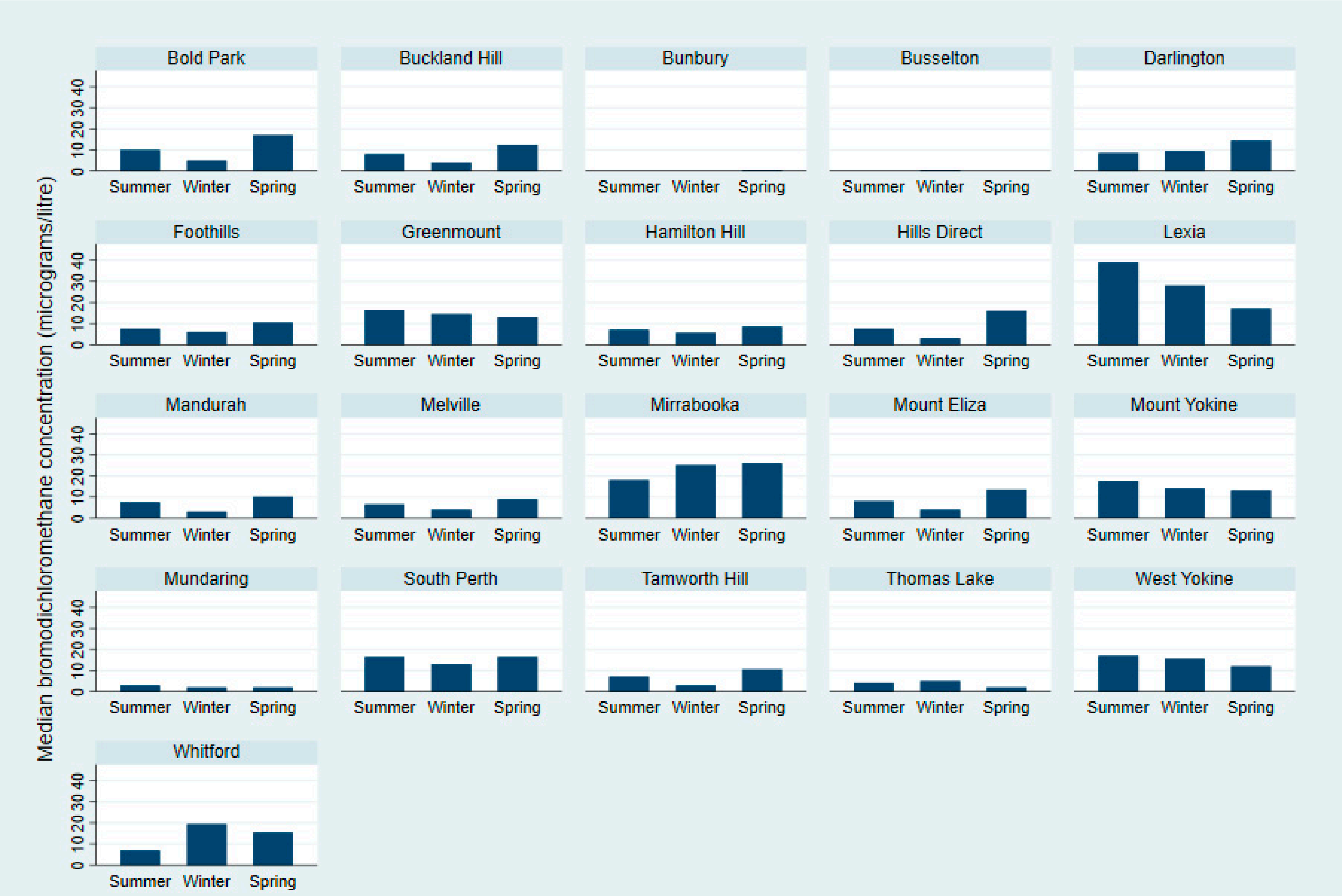

Figure S5: Seasonal variability in the median bromodichloromethane concentrations (µg/L) by water distribution zone

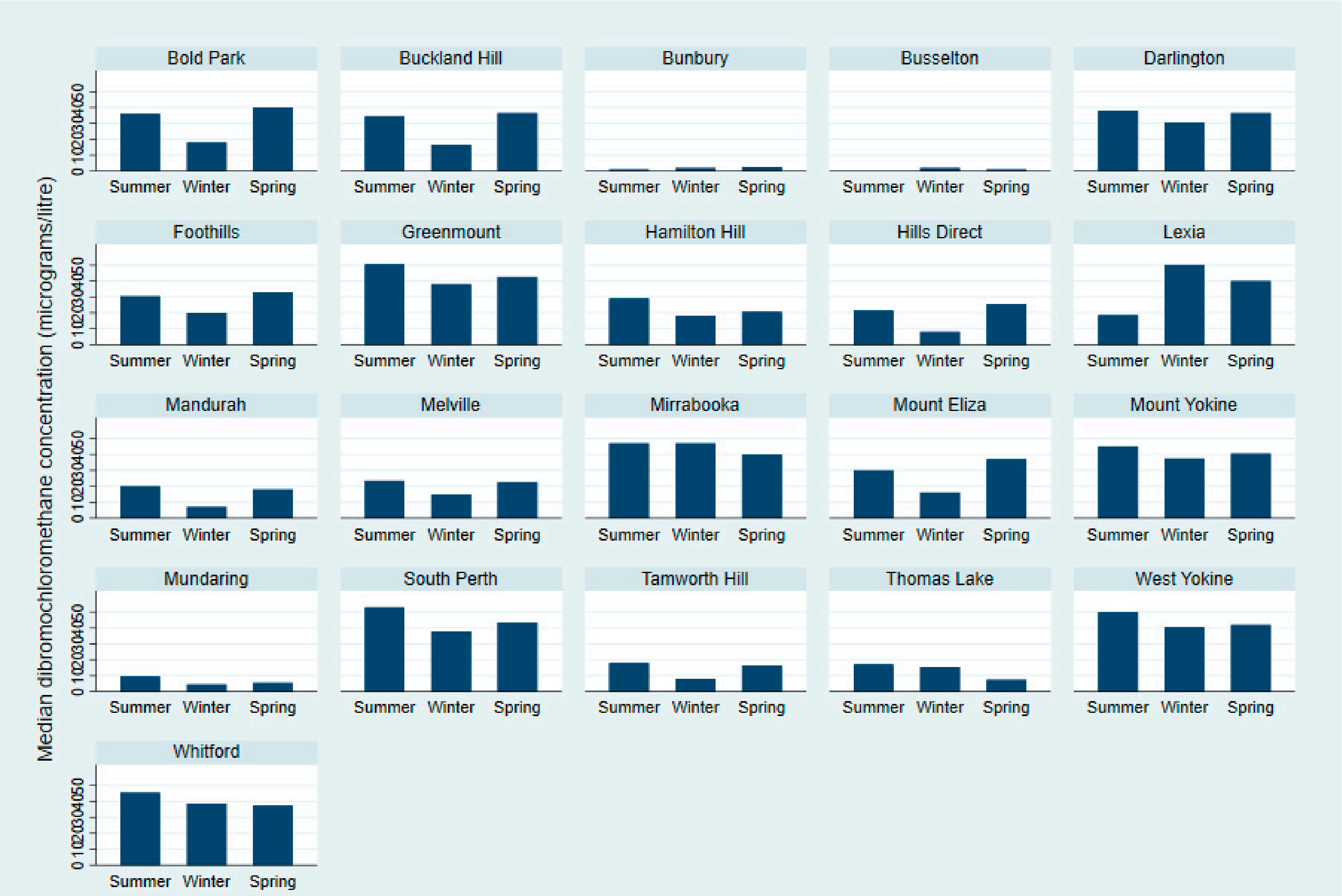

Figure S6: Seasonal variability in the median dibromochloromethane concentrations (µg/L) by water distribution zone

Supplementary Material C: Variability of trihalomethane concentrations within water distribution zones

Table S2: Variability of trihalomethane concentrations within water distribution zones

Water distribution zone	Year, Season	Site(s)	Concentration range (µg/L)					
			Total trihalomethanes	Total brominated trihalomethanes	Chloroform	Bromoform	Bromodichloromethane	Dibromochloromethane
Bold Park	2012, Summer	A	102	99	3	50	11	38
	2012, Winter	A	46	45	1	24	5	16
	2012, Spring	A	70	68	2	34	7	27
	2013, Summer	A	88	88	0	45	9	34
	2013, Winter	A	68	67	1	42	5	20
	2013, Spring	A	119	110	9	30	27	53
Buckland Hill	2012, Summer	A	111	109	2	68	8	33
	2012, Winter	A	58	57	1	36	4	17
	2012, Spring	A	79	78	1	49	5	24
	2013, Summer	A	104	104	0	60	8	36
	2013, Winter	A	55	54	1	34	4	16
	2013, Spring	A	125	119	6	50	20	49
Bunbury	2012, Summer	A,B,D	0-6	0-6	0	0-5	0	0-1
	2012, Winter	A,D,E	2-8	2-8	0	2-6	0	0-2
	2012, Spring	A,B,C,D	6-30	6-30	0-1	4-18	0-4	2-10
	2013, Summer	A,B,C,D	3-30	3-30	0	3-17	0-3	0-11
	2013, Winter	A,B,C,D	5-55	5-55	0-2	3-42	0-7	2-18
	2013, Spring	A,B,C,D	6-30	6-30	0	3-20	0-2	2-8
Busselton	2012, Summer	C,E	0	0	0	0	0	0
	2012, Winter	A,B,D,E	4-16	4-16	0	3-10	0-2	1-4
	2012, Spring	A,E	2-3	2-3	0	2	0	0-1
	2013, Summer	A,E	1	1	0	1	0	0
	2013, Winter	A,E	5-6	5-6	0	2-3	1	2
	2013, Spring	A,E	4	4	0	3	0	1
Darlington	2012, Summer	A	122	120	2	76	8	36
	2012, Winter	A	77	75	2	36	9	30
	2012, Spring	A	98	96	2	60	6	30
	2013, Summer	A	115	115	0	66	9	40
	2013, Winter	A	87	85	2	44	10	31
	2013, Spring	A	102	94	8	28	23	43
Foothills	2012, Summer	A,B	98-140	95-127	3-13	35-47	10-34	38-58
	2012, Winter	A,B	51-66	50-64	1-2	26-37	5-6	19-21
	2012, Spring	A,B	87-95	85-93	2	50-59	7	27-28
	2013, Summer	A,B	0-83	0-83	0	0-55	0-5	0-23
	2013, Winter	A,B	50-83	49-81	1-2	25-43	6-9	18-29
	2013, Spring	A,B	89-99	85-88	4-11	19-33	14-26	38-43

(continued)

Water distribution zone	Year, Season	Site(s)	Concentration range (µg/L)					
			Total trihalomethanes	Total brominated trihalomethanes	Chloroform	Bromoform	Bromodichloromethane	Dibromochloromethane
Greenmount	2012, Summer	A,B	123-157	117-147	6-10	54-65	18-24	45-58
	2012, Winter	A,B	98-101	94-96	4-5	36	17	41-43
	2012, Spring	A,B	111-118	108-125	3	51-53	13-14	44-48
	2013, Summer	A,B	126-127	123-124	3	58	15	50-51
	2013, Winter	A,B	95-104	92-101	3	49-54	11-12	32-35
	2013, Spring	A,B	112	109	3	55-56	13	40-41
Hamilton Hill	2012, Summer	A,B	79-104	78-103	1	48-70	6	24-27
	2012, Winter	A,B	51-67	50-65	1-2	28-39	5-6	17-20
	2012, Spring	A,B	48-75	47-73	1-2	28-48	4-5	15-20
	2013, Summer	A,B	82-110	81-108	1-2	42-59	8-10	31-39
	2013, Winter	A,B	55-72	53-70	2	32-45	5-6	16-19
	2013, Spring	A,B	49-66	44-59	5-7	11-16	12-16	21-27
Hills Direct	2012, Summer	A,B,C,D	45-91	43-84	2-7	23-29	5-17	15-38
	2012, Winter	A,C,D	21-43	20-42	1	10-20	2-5	8-17
	2012, Spring	A,C,D	34-87	32-81	2-6	13-22	5-18	14-41
	2013, Summer	A,C,D	34-83	34-80	0-3	15-24	4-16	15-40
	2013, Winter	A,C,D	20-46	19-44	1-2	10-23	3-6	6-15
	2013, Spring	A,C,D	53-112	47-100	6-12	10-22	14-28	23-50
Lexia	2012, Summer	A	115	64	51	2	41	21
	2012, Winter	A	142	130	12	38	33	59
	2012, Spring	A	107	103	4	45	14	44
	2013, Summer	A	116	54	62	1	37	16
	2013, Winter	A	97	87	10	23	23	41
	2013, Spring	A	89	81	8	25	20	36
Mandurah	2012, Summer	A,B,C	0-56	0-52	0-4	0-18	0-11	0-23
	2012, Winter		NA	NA	NA	NA	NA	NA
	2012, Spring	A,B,C	0-17	0-16	0-1	0-7	0-3	0-6
	2013, Summer	B,C,NR	31-49	31-47	0-2	9-16	5-9	17-22
	2013, Winter	B,C,NR	18-24	17-23	1	7-10	3-4	7-9
	2013, Spring	B,C,NR	67-77	60-68	7-9	13-14	17-21	30-34
Melville	2012, Summer	A,B	59-109	57-106	2-3	29-58	7-10	21-38
	2012, Winter	A(x2),B	22-114	21-109	1-5	11-47	2-17	8-45
	2012, Spring	A,B	43-74	41-72	2	16-44	6-7	18-22
	2013, Summer	A,B	43-87	43-87	0	18-56	5-6	19-26
	2013, Winter	A,B	21-56	20-55	1	10-36	3-4	7-15
	2013, Spring	A,B	56-103	49-99	4-7	11-57	11-15	23-31
Mirrabooka	2012, Summer	A,B	99-157	92-153	4-7	39-76	17-19	36-58
	2012, Winter	A,B	106-115	97-106	9	28-31	24-26	45-49
	2012, Spring	A,B	82-133	79-124	3-9	33-36	10-30	33-61
	2013, Summer	A,B	83-153	81-148	2-11	22-53	10-26	33-69
	2013, Winter	A,B	72-128	65-118	7-10	18-28	17-32	30-58
	2013, Spring	A,B	77-155	70-128	7-27	17-22	17-51	31-60

(continued)

Water distribution zone	Year, Season	Site(s)	Concentration range (µg/L)					
			Total trihalomethanes	Total brominated trihalomethanes	Chloroform	Bromoform	Bromodichloromethane	Dibromochloromethane
Mount Eliza	2012, Summer	A(x2),B	105-130	103-127	2-3	67-81	8-14	28-32
	2012, Winter	A(x2),B	50-79	49-79	0-1	29-68	2-4	9-16
	2012, Spring	A,B	74-87	72-85	2	43-54	6-7	23-24
	2013, Summer	A,B	84-102	84-102	0	47-65	7	30
	2013, Winter	A,B	65-108	64-105	1-3	45-56	4-12	15-37
	2013, Spring	A,B	128-151	122-145	6	52-71	20-21	50-53
Mount Yokine	2012, Summer	A,B	122-137	113-128	9	48-56	21-22	44-50
	2012, Winter	A,B	98-99	94	4-5	35	17	42
	2012, Spring	A,B	102-111	99-108	3	46	12-14	41-48
	2013, Summer	A,B	101-115	98-112	3	44-52	13-14	41-46
	2013, Winter	A,B	98-99	95-96	3	51-52	11	33
	2013, Spring	A,B	108-110	105-107	3	53-54	13	39-40
Mundaring	2012, Summer	A	31	30	1	15	4	11
	2012, Winter	A	15	14	1	8	2	4
	2012, Spring	A	14	14	0	7	2	5
	2013, Summer	A	21	21	0	11	2	8
	2013, Winter	A	16	16	0	9	2	5
	2013, Spring	A	15	15	0	7	2	6
South Perth – Kewdale	2012, Summer	A,B,C	131-145	122-135	8-10	47-56	19-22	54-57
	2012, Winter	A(x2),B,C	69-155	67-149	2-6	36-78	7-21	24-52
	2012, Spring	A,B,C	76-123	71-119	3-5	19-59	13-16	36-47
	2013, Summer	A,B,C	56-131	56-128	0-3	26-62	7-14	23-52
	2013, Winter	A,B,C	109-115	106-112	3	58-65	10-13	31-38
	2013, Spring	A,B,C	76-116	68-111	5-9	14-50	17-25	34-49
Tamworth Hill	2012, Summer	A,B,C	43-46	41-44	2	14-16	8-10	18-20
	2012, Winter		NA	NA	NA	NA	NA	NA
	2012, Spring	A,B,C	3-17	3-16	0-1	2-9	0-3	1-4
	2013, Summer	A,B,C	29-37	29-37	0	9-13	5-6	15-18
	2013, Winter	A,B,C	19-21	18-20	1	7-9	3	8
	2013, Spring	A,B,C	67-71	59-63	8	12-13	18-19	29-31
Thomson's Lake	2012, Summer	A,B	49	48-49	0-1	32-34	3	12-13
	2012, Winter	A,B	47-49	45-47	2	25-26	5	15-16
	2012, Spring	A,B	41-43	41-42	0-1	27	3	11-12
	2013, Summer	A,B	63-83	62-81	1-2	36-46	5-8	21-27
	2013, Winter	A,B	45-67	43-63	2-4	24-35	5-8	14-20
	2013, Spring	A,B	8-15	8-15	0	4-10	1	3-4
West Yokine	2012, Summer	A,B	131-152	123-143	8-9	54-65	20-23	49-55
	2012, Winter	A,B	98-105	94-100	4-5	35-38	17-18	42-44
	2012, Spring	A,B	97-105	94-102	3	44-48	11-12	39-42
	2013, Summer	A,B	108-130	105-127	3	49-62	13-14	43-51
	2013, Winter	A,B	102-111	99-107	3-4	52-54	12-14	35-39
	2013, Spring	B	141	132	9	55	24	53

(continued)

Water distribution zone	Year, Season	Site(s)	Concentration range (µg/L)					
			Total trihalomethanes	Total brominated trihalomethanes	Chloroform	Bromoform	Bromodichloromethane	Dibromochloromethane
Whitford	2012, Summer	A	111	103	8	48	2	53
	2012, Winter	A	112	105	7	34	23	48
	2012, Spring	A	96	93	3	44	11	38
	2013, Summer	A	93	91	2	41	12	38
	2013, Winter	A	69	63	6	18	16	29
	2013, Spring	A	88	81	7	24	20	37

Notes: NR: not recorded. NA: not applicable as no sampling occurred for that season. Blue (light) shading indicates a range of ≤30 µg/L, orange (dark) shading indicates a range of >30 µg/L within a site in the same season, and no shading indicates that sampling only occurred at one site.
